# Computational Investigation of the Potential and Limitations of Machine Learning with Neural Network Circuits Based on Synaptic Transistors

**DOI:** 10.1021/acs.jpclett.4c01413

**Published:** 2024-06-28

**Authors:** Sergei Manzhos, Qun Gao Chen, Wen-Ya Lee, Yoon Heejoo, Manabu Ihara, Chu-Chen Chueh

**Affiliations:** † School of Materials and Chemical Technology, Tokyo Institute of Technology, Ookayama 2-12-1, Meguro-ku, Tokyo 152-8552, Japan; ‡ Department of Chemical Engineering and Biotechnology, National Taipei University of Technology, Taipei 106, Taiwan; § Department of Chemical Engineering, National Taiwan University, Taipei 10617, Taiwan

## Abstract

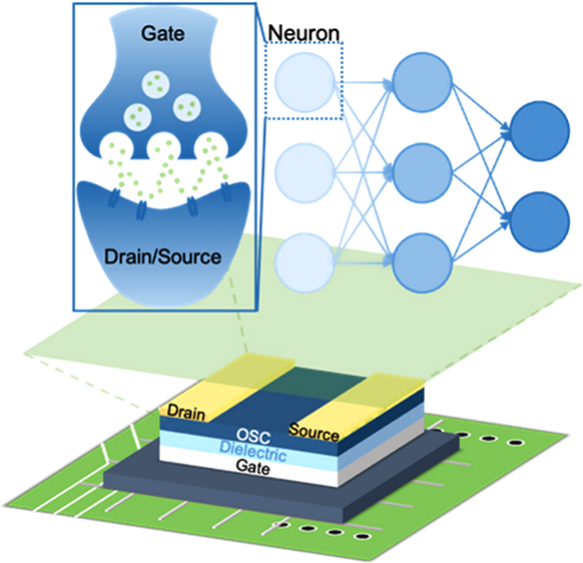

Synaptic transistors have been proposed to implement neuron activation functions of neural networks (NNs). While promising to enable compact, fast, inexpensive, and energy-efficient dedicated NN circuits, they also have limitations compared to digital NNs (realized as codes for digital processors), including shape choices of the activation function using particular types of transistor implementation, and instabilities due to noise and other factors present in analog circuits. We present a computational study of the effects of these factors on NN performance and find that, while accuracy competitive with traditional NNs can be realized for many applications, there is high sensitivity to the instability in the shape of the activation function, suggesting that, when highly accurate NNs are required, high-precision circuitry should be developed beyond what has been reported for synaptic transistors to date.

Machine learning (ML) using neural networks (NNs) has acquired significant importance in science and technology.^1^ The universal approximator properties^2,3^ of neural networks combined with their generality, i.e., the ability to be trained to perform complex input–output mappings, make them a versatile approach applicable in very heterogeneous fields from social sciences to computational chemistry.^4−7^ In most applications, NNs are realized as codes for digital computers. In some applications, the NNs are computationally intensive due to the large number of neurons in the network, the size, dimensionality, and complexity of the data set, or both. Examples of large, computationally intensive NNs are NNs used in speech or complex image analysis. In some applications, speech and image analysis included, one can pretrain a large NN on a large amount of data and use it in an application without retraining.

CPU and energy costs of calling an NN model, as well as the cost, weight, and size of the device realizing the NN, can be critically important. Examples of such applications include portable applications such as wearable sensors monitoring the state of health of living organisms or machines, where a digital CPU-based NN could be unwieldy. Uses in remote locations or extreme environments (e.g., in air or space, under-water) are also examples of applications where a small, cheap, easily replaceable, and robust device implementing a pretrained NN is preferred over a digital computer. The speed of computation with an NN can also be important. From the analysis of rapidly changing scenes to high-frequency trading, the speed of computation may be a limiting factor of the utility of an NN. Recognizing these issues, the industry has been developing NPUs (neural processing units).^8,9^ Although purposed, these devices are still digital computers and require all the infrastructure of a digital CPU around them.

In such applications, a dedicated analog electronic circuit has advantages as it could achieve simultaneously low cost, low weight and size, low energy consumption, and high computation speed. Optical circuits could also potentially be used for this purpose (and address the issue of computation speed at its root by performing computations at the speed of light),^10,11^ but achieving optical nonlinearity at small light intensities remains an outstanding problem;^12−14^ feasible NN architectures (neuron connectivity patterns) are also limited.^15^ On the contrary, semiconductor circuits can rely on available, ready-to-use technologies used in the vast semiconductor electronics industry. The function of neurons of an NN can be played by individual transistors or their circuits (that could realize more complex shapes of neuron activation functions than individual transistors). The parameters of the NN (weights and biases) can be pretrained on a digital computer and implemented via resistive circuits. In principle, different neurons can have different NAFs (neuron activation functions) within those realizable by the transistor types used. In recent years, synaptic transistors, both inorganic and organic, realizing nonlinear responses suitable for use as NAFs in NNs, have been actively researched.^16−21^ Several groups have mimicked the behavior of human synaptic signals through gate voltage stimulation. Lee’s group improved the nonvolatility of artificial synapses by modifying the side chains of conjugated polymers.^16^ Some of us explored ambipolar conjugated polymer transistors to study artificial synaptic behavior.^22^ In addition, we studied the active channel with ionic dopants to mimic synaptic behavior in artificial synapses.^23^

NNs based on synaptic transistors have, however, specific challenges. On a digital computer, it is easy to realize any shape of the neuron activation function. In regression type NNs, while one and the same predetermined activation function (such as the often-used sigmoid function) for all neurons can be used to approximate any smooth function, there are significant advantages of using purpose-specific NAFs. Any smooth nonlinear function can play the role of a neuron activation function.^3^ For example, exponential neurons can be used to build some-of-product representations useful in multidimensional integration.^24^ Moreover, the shapes of neuron activation functions can be made individual to each neuron. This increases the expressive power of the NN; some of us recently showed that, in this way, one can obviate the need for nonlinear parameter optimization which is largely responsible for CPU cost and overfitting.^25^ However, when using a synaptic neuron-based NN, one is limited to NAFs realized by specific types of the used transistors or their superpositions.

An important issue specific to analog circuit-based NNs is noise and other instabilities. The available literature on synaptic transistor-based NN reported substantial instabilities of the NAF shape from experiment to experiment.^17,19,20^ Chen’s group utilized optoelectronic synaptic transistor-based NNs to impose noise on the training data during the training process.^19^ Lee’s group introduced Gaussian-distributed random noise into the synaptic weights during the training process of spike neural networks (SNNs).^20^ That is, while there have been studies of the effect of perturbation to NN inputs and weights (corresponding to instabilities or noise in the resistive circuit around the transistor), the effect of the factor of instability of the shape of the NAF itself on NN performance, to the best of our knowledge, remains unquantified even though, as we demonstrate in this article, it appears to be critically important.

In this work, we therefore study the effect on the quality of machine learning of limiting the type of shapes of NAFs to those realizable with transistors, while allowing different neurons to have different NAFs. We compare the accuracy of the NN to an NN with optimal NAFs for a given ML problem. We also study the effect of perturbations of the shape of NAFs. We use 2 representative ML problems: (i) machine learning of molecular potential energy surfaces (PES) on the example of the PES of H_2_O, as an example where very high accuracy is required and obtainable, and where the ML problem is well understood from prior works;^25,26^ (ii) machine learning predictions of material properties where there is potential to use dedicated circuits due to the vastness of compositional and structural space that can then be searched more efficiently. The concept and structure of this article are summarized in Figure 1.

**Figure 1:**
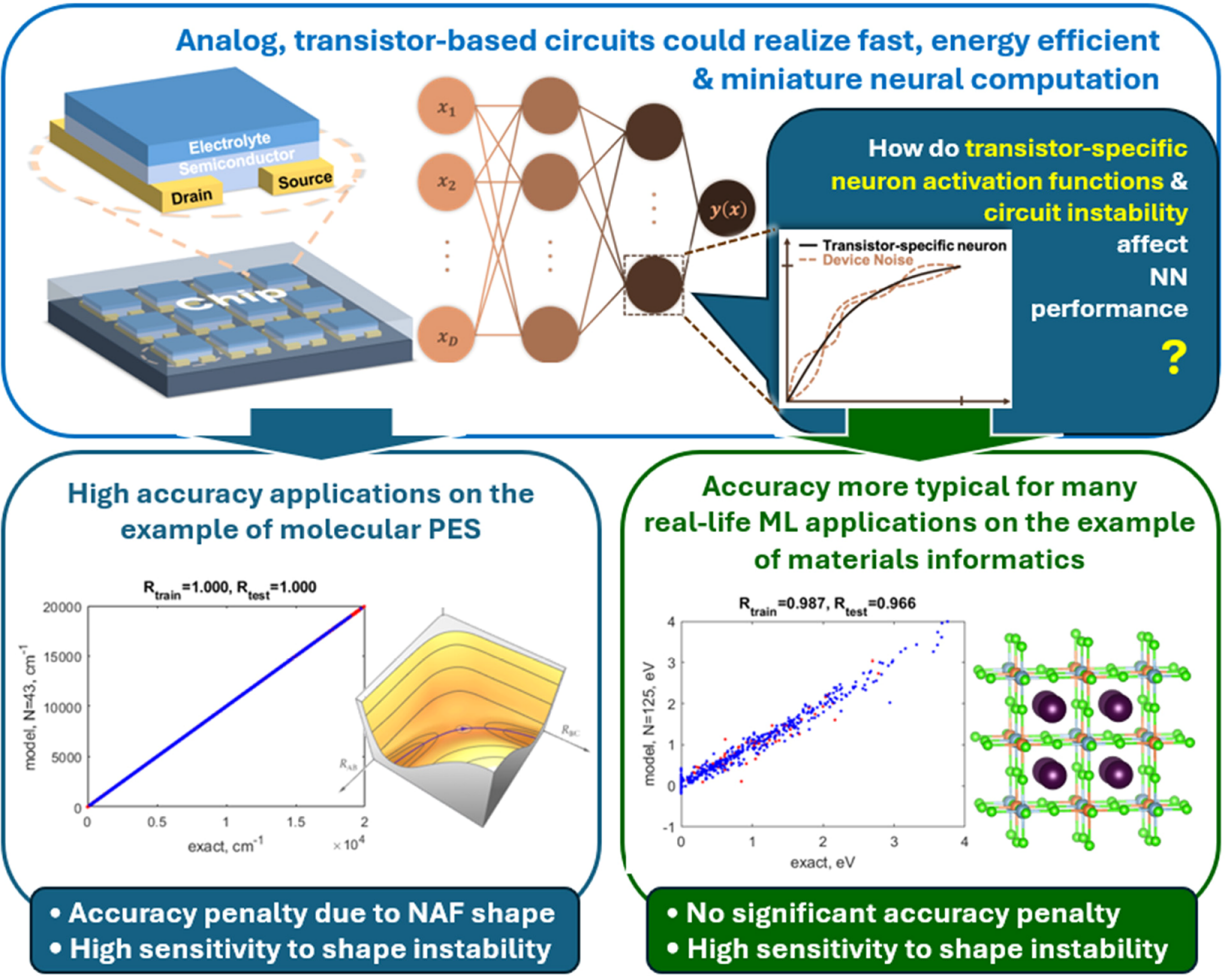
Concept and the structure of this work. We investigate the effects on NN performance of limiting the NN neuron activation function (NAF) shapes to those typically obtained with transistors and of perturbation to the shape of NAF due to circuit instability and noise that are expected in real-life analog NN circuits. We consider the effect of these factors in a high accuracy regime on the example of fitting a small-molecule potential energy surface (PES) and an accuracy regime more typical of most real-life machine learning applications on the example of predicting the bandgaps of double perovskites A_2_B^1+^B^3+^X_6_.

We use the method of Manzhos and Ihara that effectively implements a single hidden layer neural network with optimal neuron activation functions for each neuron built with kernel regression.^25^ The reader is referred to ref (25) for details; here, we only briefly summarize the method. The method builds a single-hidden layer NN representation of the target function *f*(***x***), where ***x*** ∈ *R*^*D*^ is a vector of inputs (descriptors),
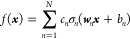
1as
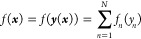
2where *y*_*n*_ = ***w*_*n*_*x***, i.e., ***y*** = ***Wx***, ***y*** ∈ *R*^*N*^ with a rectangular *N* × *D* matrix ***W*** (whose rows are vectors ***w*_*n*_**), and where univariate functions *f*_*n*_(*y*_*n*_) (that combine the neuron activation functions and their output weights) are built with kernel regression:
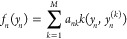
3where *M* is the number of training data points and *k*(*y*, *y*′) is the kernel. That is, we construct an NN with flexible and different for each neuron shapes of the activation functions. In eq 1, we omit the output neuron without the loss of generality. The definition of ***y*** in eq 2 also omits biases (cf. eq 1) because their effect can be subsumed into the shape of *f*_*n*_(*y*_*n*_). In ref (25), it was shown that the coefficients *a*_*nk*_ for all *f*_*n*_ at once can be obtained with Gaussian process regression (GPR)^27^ or kernel ridge regression (KRR)^28^ by using a first order additive kernel in ***y***.^29,30^ In this way, neuron activation functions that are optimal for a given ML problem (data set) and for a given weights matrix ***W*** are built for each neuron. This allows obviating nonlinear fitting of ***W*** and thereby saving the CPU cost and alleviating the problems of overfitting and local minima that are associated with nonlinear parameter fitting. Some of us showed in refs (25 and 31) that highly accurate machine learning can be realized by using ***W*** set by rules. We use the same rules to form ***W*** as in refs (25 and 31), namely, the rows of ***W*** are elements of a *D*-dimensional Sobol sequence. The method builds a representation equivalent to a single hidden layer NN with the help of additive kernel regression (we can call this representation GPR-NN). While, in many applications, multilayer (deep) NNs are used, a single hidden layer NN is a universal approximator.^3,32,33^ It is sufficient for the present purpose of studying the effects of restrictions in the shape of and perturbations to the NAFs.

If the kernel used in eq 3 possesses sufficiently high expressive power, such as the commonly used RBF kernel (), then eq 3 in principle allows constructing any shape of the neuron activation function and therefore finding the optimal shape. In dedicated circuits realizing NNs based on transistor circuits, however, possible forms of the neuron activation functions are limited to those obtainable with one or a small number of transistors. Those can be most generally described with functions of the form *y*(*x*) = *A*(1–*e*^–*Bx*^).^17,20^ On the other hand, within this restriction, there is no requirement for all neurons of the NN to have the same neuron activation functions, and one should optimize those for each neuron, which can be done on a digital computer (e.g., with the method of ref (25)) and then implemented on the transistor circuit.

The restrictions on possible shapes of the activation function can be implemented by the choice of the additive kernel. It was previously demonstrated that product kernels allow obtaining polynomial approximations.^34,35^ Kernels of physically motivated shapes have been used in the RKHS (reproducing kernel Hilbert space) method^36,37^ that is used, in particular, to fit molecular potential energy surfaces (PESs). Here, we use an exponential product kernel
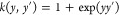
4to limit the shapes of *f*_*n*_ to those constructed from typical transfer functions obtained with transistors while allowing them to be different for different *n*. Note that coefficients before the summands in eq 4 are immaterial as they are fully accounted for by *a*_*nk*_.^38^ To study the effect of instability of the activation function on NN performance, we apply random perturbations to *f*_*n*_(*y*_*n*_) whereby the perturbed function is *f*_*n*_′(*y*_*n*_) = *f*_*n*_(*y*_*n*_)(1 + *Ar*), where *A* is noise amplitude and *r* is a uniformly distributed random number on [−0.5, 0.5].

We consider two applications: first, we fit a potential energy surface of the water molecule as an example of an application which is well understood, where high accuracy of machine learning is required and obtainable and where possible accuracies with different machine learning algorithms are well-known from prior research to which the present results can be compared. This is not an application where a dedicated NN circuit is required, but we use it to study in a controlled way the effects of limitations and perturbations of the shape of the activation function. In particular, the data set permits using a large test set (much larger than the training set) to reliably evaluate the quality of the approximations, which is often not possible in real life applications where data are few. Next, we consider a materials informatics application, where achievable accuracy is lower and where available data are relatively few. ML for materials informatics is done today on digital computers, but there are potential advantages of employing dedicated circuits to sieve through astronomical numbers of possible compositions and structures of materials or molecules.

The calculations are performed in Matlab with a modified version of the code provided in ref (25); the code and the data are provided as Supporting Information. The modification concerned direct implementation of GPR equations (for simplicity and to facilitate the application of perturbations to the activation functions) instead of using Matlab’s *fitrgp* function and implementation of the kernel of eq 4.

We start by fitting the data set sampling an analytical PES of H_2_O in Radau coordinates (i.e., three input features) in the spectroscopically relevant region up to 20,000 cm^–1^ above the equilibrium geometry. The reader is referred to refs (25, 26, and 39) for the description of the data (the data are also available for download from those references). We fit 1000 randomly selected points and test the quality of the model on a large set of 9,000 test points. In this application, the desired level of PES accuracy to provide a spectroscopically accurate PES is on the order of 1 cm^–1^. With an isotropic RBF kernel (length parameter *l* = 0.45 and inputs scaled to unit cube) and noise parameter (used when computing the coefficients *a*_*nk*_ via kernel regression) σ^2^ = 10^–12^ (we report the results with hyperparameters optimized for the best test set error), one obtains a sub-cm^–1^*test* set error irrespective of a particular random draw of train and test points with *N* = 40 or more neurons. The reader is referred to ref (25) for an example of the trend of train and test set errors as a function of the number of neurons achieved with this method on the same data as well as for a comparison to a traditional NN. Figure 2 shows that the resulting NN (with *N* = 40) obtained a highly accurate model with correlation coefficients between exact and predicted data of 1.000 for both training and test set points. Figure 2 also shows the shapes of the activation functions for the largest terms in eq 2. The magnitude of these terms is also plotted in the figure; the difference in their magnitude could be used to evaluate the relative importance of features or to discard unimportant features or coupling terms; see refs (25 and 40) for examples of such uses. The optimal shapes are different between neurons and are of various forms. In particular, they are not restricted to monotonic functions typically used in NNs (such as the often-used sigmoid or ReLU type functions). For the particular case shown in the figure, the training/test set errors are 0.25/0.63 cm^–1^ (ranging (0.24–0.26)/(0.51–0.75) depending on the particular random draw of points, based on 10 runs). These numbers can be compared to previous machine learning results for the same PES. In ref (26), a traditional NN with sigmoid NAFs was used by some of us to fit this PES to a spectroscopic accuracy for the first time, whereby a test set rmse of 1 cm^–1^ was obtained when fitting 1500 points, using 23 neurons. It was also concluded in that work that the sigmoid activation function is the best. Much larger test set rmse’s were reported with NNs in other works albeit using fewer training points.^41,42^ In ref (31), a test set error of 0.57 cm^–1^ was obtained fitting 1000 points with the same method as used here (i.e., GPR-NN). In ref (39), a GPR model obtained a test set error of 1.9 cm^–1^ fitting 500 points.

**Figure 2:**
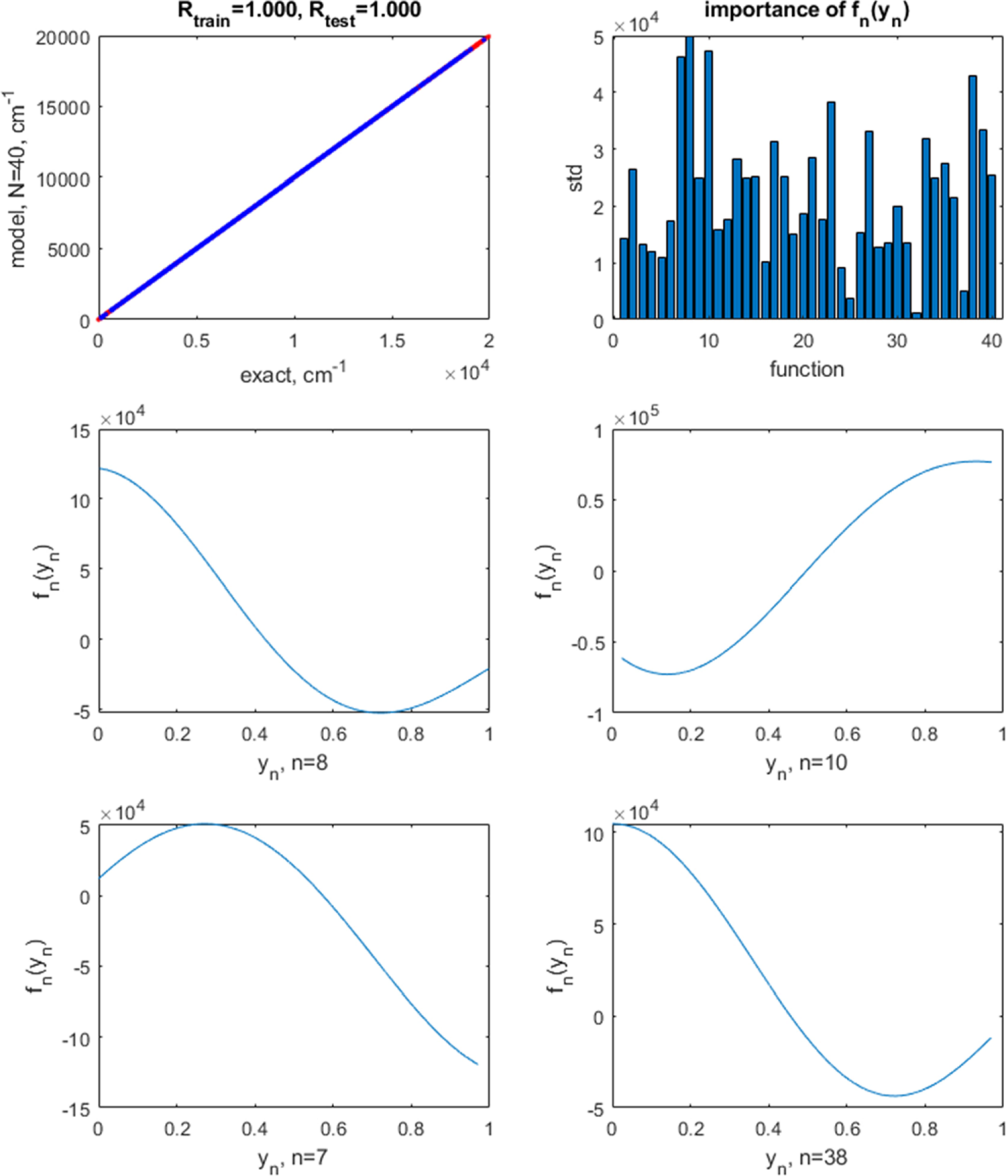
Top row: the correlation plot between the exact and predicted values of the potential energy of H_2_O when using a neural network with *N* = 40 optimal (built with RBF kernels) neuron activation functions (left) and the magnitudes of terms *f*_*n*_(*y*_*n*_) by their standard deviation (std). The blue points are for training and red points, for test set data. In this case, they visually overlap because of the high accuracy of the model. The correlation coefficients *R* are also given on the plot separately for training and test sets. The following 4 panels show the shapes of *f*_*n*_(*y*_*n*_) for the largest magnitude terms.

The result of restricting the shapes of *f*_*n*_(*y*_*n*_) to those derived from eq 4 is shown in the same format in Figure 3 for the same number of neurons (*N* = 40). Figure 3 also shows the shapes of the activation functions for the largest terms–one can see that, due to the use of the exponential product kernel, they are indeed restricted to those of the type obtainable with transistors. The optimal noise parameter with this kernel is lower, σ^2^ = 10^–13^. While visually the quality of the fit remains very high (correlation coefficients of 1.000), the train/test errors are significantly higher, ranging (1.49–2.01)/(2.43–2.85) depending on the particular random draw of points (based on 10 runs). That is, there is an accuracy penalty for restricting the neuron activation function shapes to those obtainable with synaptic transistors. This penalty can be *partially* compensated by using (substantially) more neurons: for example, with 100 neurons, we obtain train/test set errors ranging (0.82–1.44)/(1.24–2.36) depending on the particular random draw of points (based on 10 runs). Even with higher *N*, the errors never become as low as those achieved with the RBF kernel, i.e., with fully optimal activation functions.

**Figure 3:**
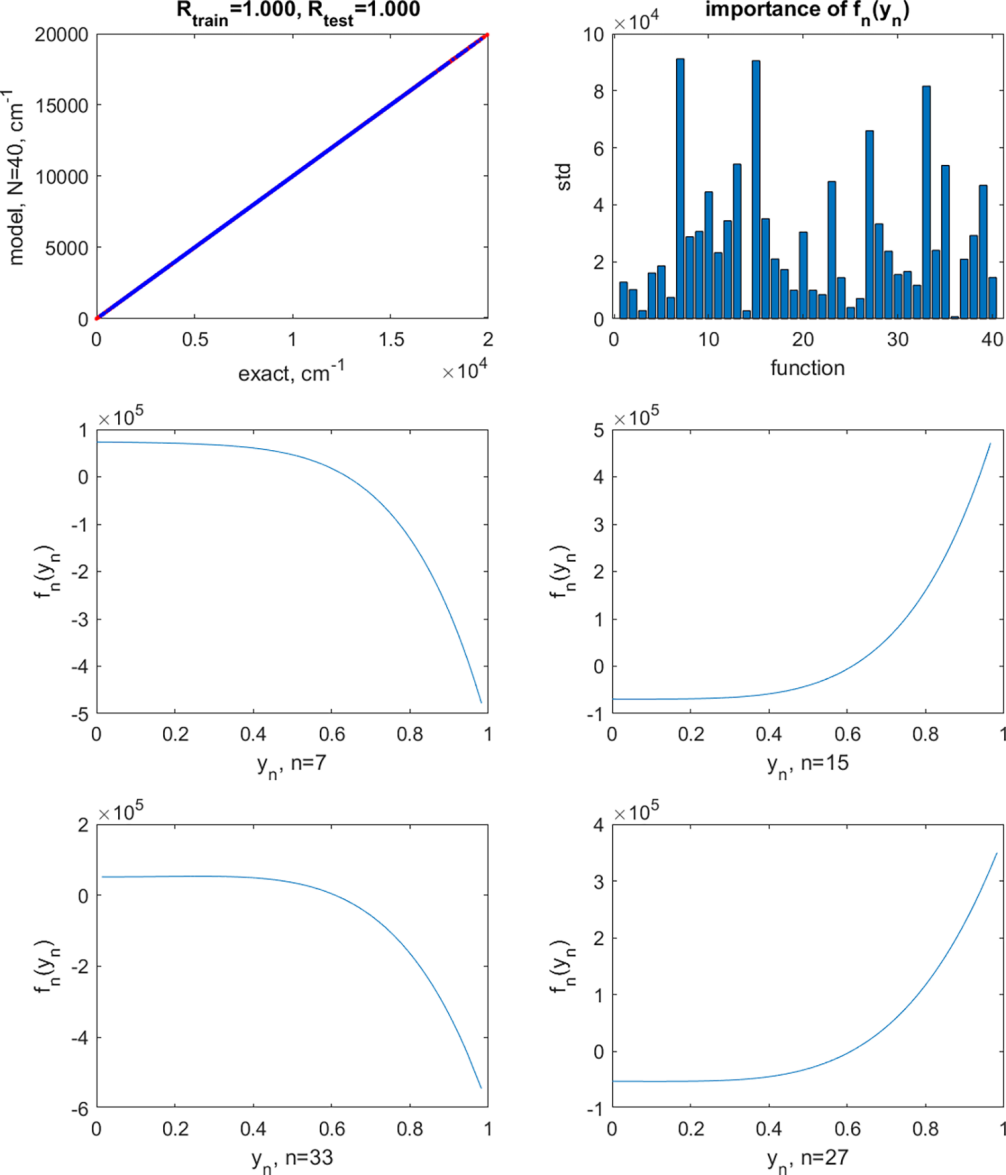
Top row: the correlation plot between the exact and predicted values of the potential energy of H_2_O when using a neural network with *N* = 40 neuron activation functions restricted to exponential shapes (left) and the magnitudes of terms *f*_*n*_(*y*_*n*_) by their standard deviation (std). The blue points are for training and red points, for test set data. In this case, they visually overlap because of the high accuracy of the model. The correlation coefficients *R* are also given on the plot separately for training and test sets. The following 4 panels show the shapes of *f*_*n*_(*y*_*n*_) for the largest magnitude terms.

It was reported before that using a different NAF may lower the achievable accuracy when doing high-accuracy fitting of a PES: in ref (24), some of us introduced the simple exponential NAF as a way to easily obtain a sum of products representation (useful or even necessary in some applications requiring multidimensional integration, in particular quantum dynamics^43^). In that case, the choice of NAF was dictated by the desired properties rather than by a NAF achievable with a particular NN here (i.e., transistor-based). When refitting the PES of HOOH with an NN using 90 neurons, the test rmse was about 10 cm^–1^ with a sigmoid NAF and about 20 cm^–1^ with an exponential NAF.^24^ We have fitted the present data also with a traditional NN using the same number (40) of either sigmoid or exponential neurons using the powerful Levenberg–Marquardt optimizer (in Matlab). We obtained train/test errors of 0.64–1.47/1.57–3.42 cm^–1^ with sigmoid and 1.47–5.68/3.10–13.62 cm^–1^ with exponential neurons (the ranges are for 5 runs with different train/test splits and NN initializations). This illustrates the general point that “non-standard” NAF shapes may come at a cost in accuracy. This also illustrates the advantage of optimal NAFs built by the GPR-NN method, even though the method does not optimize ***W***.

When applying random perturbations to the shapes of the neuron activation functions, we observe severe degradation of performance. Figure 4 shows the results with *A* = 0.1. The perturbed shapes of the activation functions shown in the figure are within the degrees of device-to-device and run-to-run variations observed with some experimental synaptic transistors.^17,19,20^ The optimal noise parameter was σ^2^ = 10^–4^. The range of train/test set errors is (1301–1375)/(1321–1367) cm^–1^ (over different train/test splits of the data). With *A* = 0.01 (optimal σ^2^ = 10^–7^), the shapes of the activation functions are visually indistinguishable from the nonperturbed ones, but the perturbation still has a strong effect on the model accuracy: The train/test set errors are (207–215)/(208–213) cm^–1^. These errors are too high, for example, for the PES to be used for the calculation of vibrational spectra. We conclude that, when highly accurate ML is required, transistor-based neuron activation functions may be disadvantaged. Alternatively, highly stable circuitry needs to be developed.

**Figure 4:**
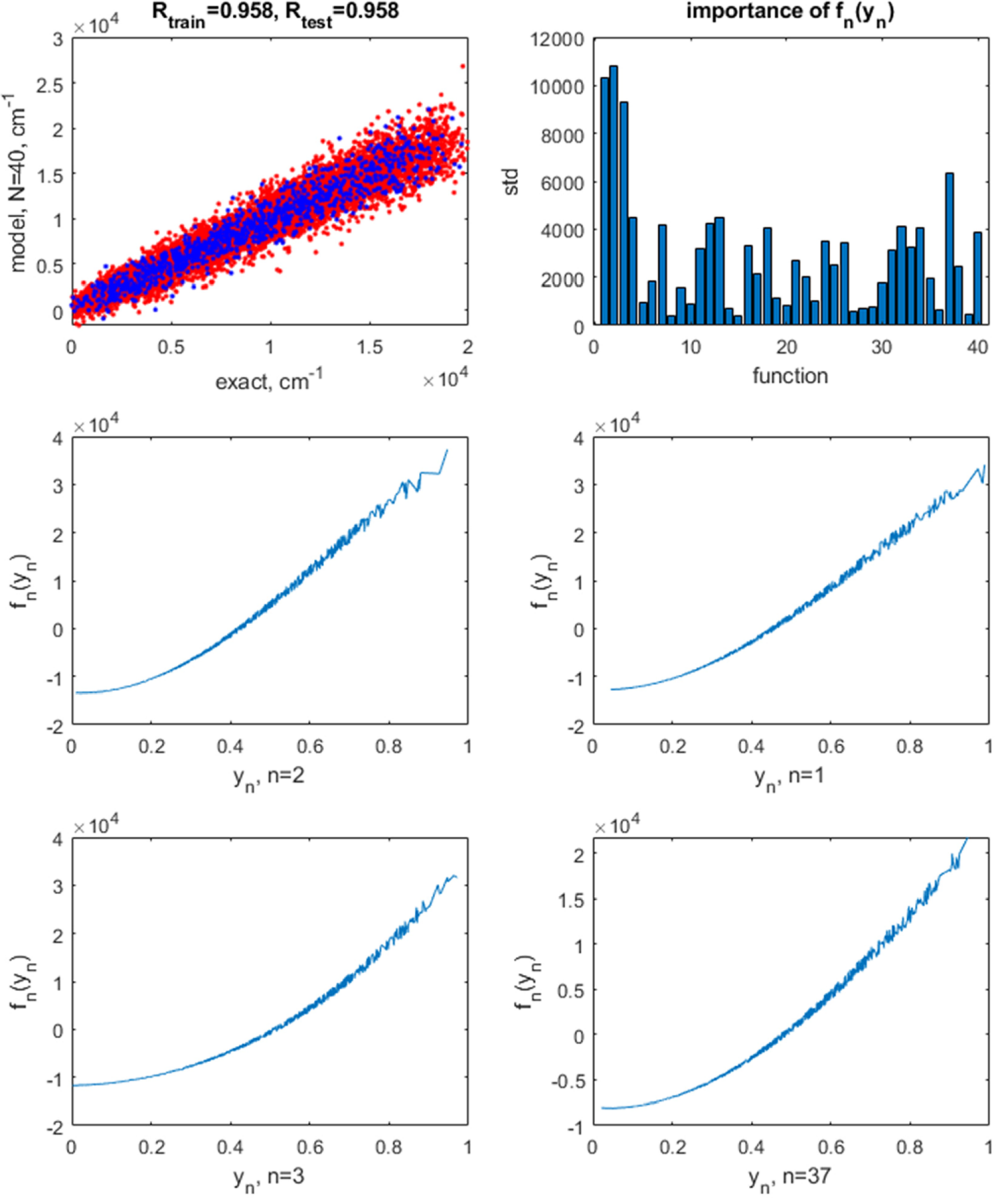
Same as Figure 3 but applying a perturbation *A* = 0.1 to the neuron activation functions.

In many applications, however, accuracy as high as in the previous example is not required or is not attainable due to the noisy nature or the scarcity of the data. An example of such an application is materials informatics. The reader is referred to the literature for typical ML accuracies attainable when predicting materials properties or device performance characteristics, where correlation coefficients between the exact and ML-predicted properties are often lower than 0.9.^44−50^

We now apply the method to machine learning of bandgaps of double inorganic perovskites A_2_B^1+^B^3+^X_6_, where A sites are occupied by alkali metal atoms Cs, K, or Rb, B^1+^ sites by Ag, Au, Cu, In, or Tl, B^3+^ sites by Al, As, Bi, Ga, In, or Sb, and X by halogen atoms (Br, Cl, or I). The method of ref (25) was recently applied to this machine learning problem and showed advantages over standard ML methods; in particular, in the accuracy of bandgap prediction in the visible region, see ref (40) for details. We use the same data set as reported in ref (51) and used also in ref (40). Briefly, the band gap is learned from 24 descriptors of composition and structure including electronegativities, ionization potentials, *s*-, *p*-, and *d*- valence orbital radii, and frontier orbital energies of the atoms at each site as well as interatomic distances. These 24 descriptors were identified in ref (40) as the most important out of the 31 numeric descriptors reported in ref (51). They are scaled on a unit cube. The reference bandgap in the data set was computed with DFT^52^ using PBE functional.^53^ The data set is small, containing only 540 structures, and here, as in many real-life applications, the luxury of using large test sets cannot be afforded. We randomly select 432 structures for training and 108 for testing (corresponding to the often-used 80/20 split). Due to the smallness of the data set, for reliable comparison between the types of neuron activation functions, we fix the random seed (see ref (40) for the effect of different train-test splits on ML accuracy with these data) when doing train-test split.

Based on the results of ref (40) with the RBF kernel, the best bandgap prediction accuracy of about 0.2 eV is achieved using about 100 or more neurons. In Figure 5, we show the results with 125 neurons built using RBF kernels (*l* = 1.0, σ^2^ = 10^–7^). The train/test set error is 0.14/0.18 eV. The optimal neuron activation functions are smooth, uncomplicated shapes. Using the same number of neurons with the kernel of eq 4, we obtain train/test errors of 0.14/0.17 eV, i.e., similar to the accuracy achieved with optimal NAFs. The correlation plots and the shapes of the resulting NAFs are shown in Figure 6. Contrary to the example of PES fitting above, *there is no accuracy penalty* due to the restrictions on the shape of the NAF, or more precisely, limitations on prediction accuracy due to the data dominate those due to the restricted shape of the activation function. We have observed a degree of sensitivity to the type of the NAFs in this case with the conventional NN: with a sigmoid NN, using 10 (15) neurons, we obtain train/test errors of 0.06–0.09/0.15–0.26 (0.03/0.15–0.33) eV. The range is for different runs with different NN parameter initializations (the train-test split is kept the same as in the GPR-NN calculations). The train/test errors are 0.06–0.08/0.19–0.65 (0.04–0.05/0.31–0.74) eV, respectively, for the exponential NN. Errors are larger for smaller NNs, and significant overfitting (i.e., larger test errors) is observed for larger NNs. This is a consequence of the small number of data points. While the lowest rmse’s are by and large comparable for a conventional NN with both NAFs, a conventional NN with exponential neurons risks a higher chance of getting stuck in a local minimum (which is what the range of errors over different runs indicates)–something that GPR-NN does not suffer from as it obviates nonlinear optimization. In this relatively lower accuracy regime, the choice of NAF is less critical (if one can avoid local minima), which can also be confirmed by using the ReLU NAF: with 15 neurons, train/test errors are 0.09–0.13/0.15–0.34 eV. The number of neurons is higher with GPR-NN than in a traditional NN, as ***W*** is not optimized, but there is no issue of local minima; adding neurons in GPR-NN does not cause a significant cost overhead (the only extra cost is the summation in the additive kernel) or overfitting, so that one does not have to search for an optimal *N*, see ref (25). The absence of the need to optimize ***W*** in the GPR-NN method can also be advantageous for transistor-based NNs as standardized resistive circuits could then be used.

**Figure 5:**
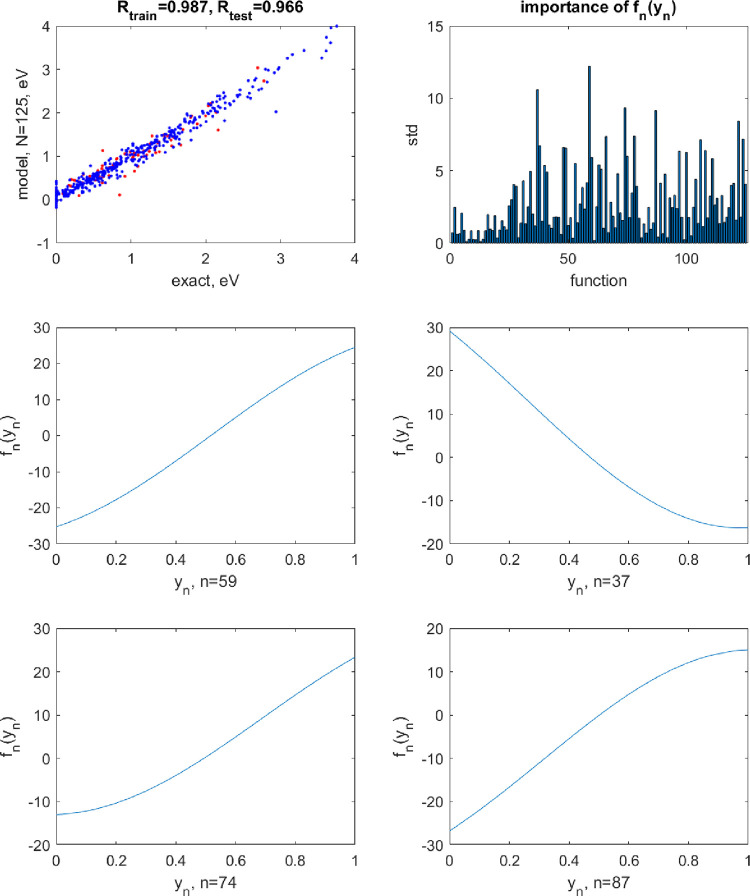
Top row: the correlation plot between the exact and predicted values of bandgap of double perovskites A_2_B^1+^B^3+^X_6_ when using a neural network with *N* = 125 neuron activation functions built with RBF kernels (left) and the magnitudes of terms *f*_*n*_(*y*_*n*_) by their standard deviation (std). The blue points are for training and red points, for test set data. The correlation coefficients *R* are also given on the plot separately for training and test sets. The following 4 panels show the shapes of *f*_*n*_(*y*_*n*_) for the largest magnitude terms.

**Figure 6:**
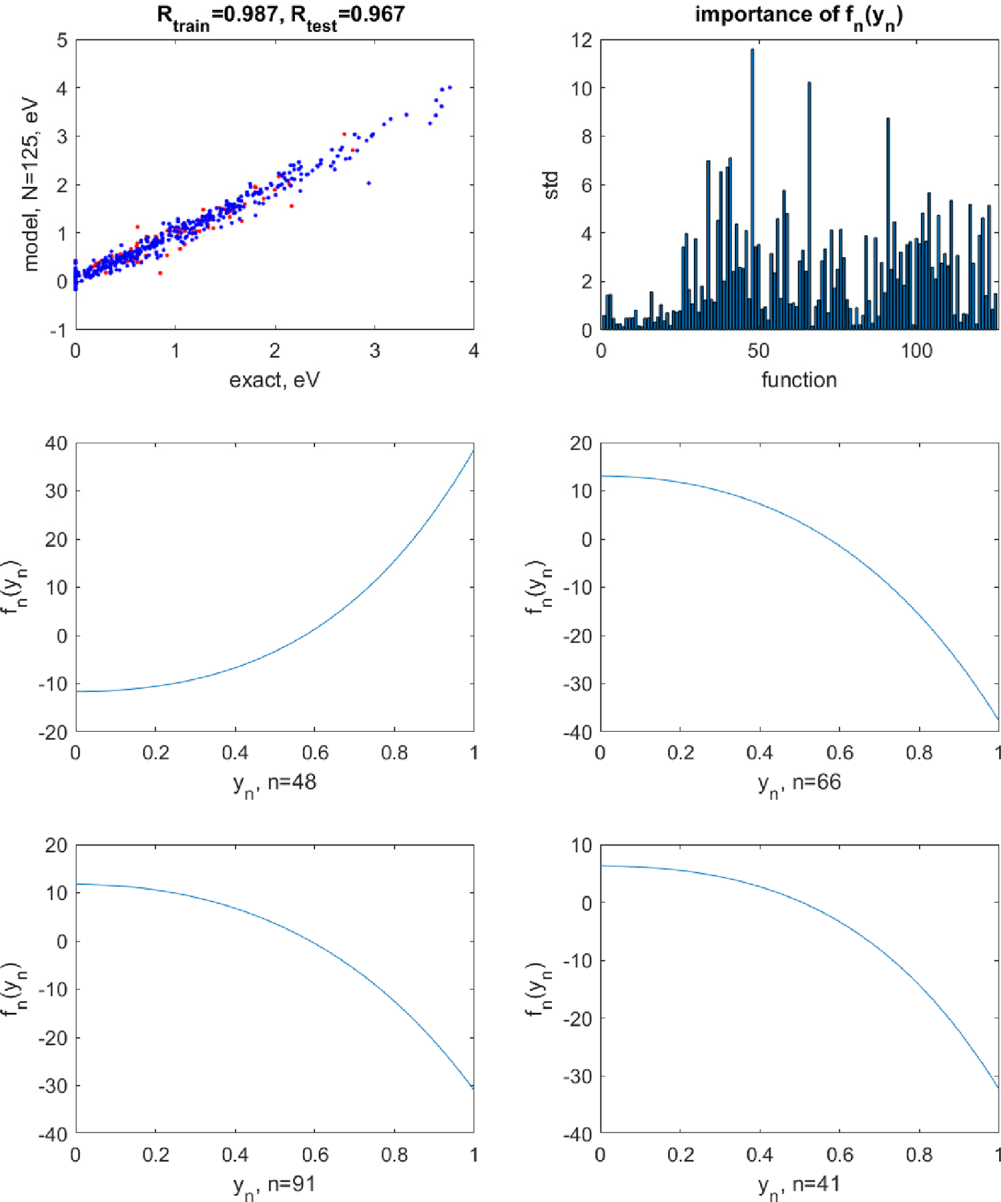
Same as Figure 5 but with the activation functions built with eq 4.

However, the sensitivity to perturbations of the shape of the NAF remains high also in this case. Figure 7 shows the results of the same NN when the activation functions are perturbed with *A* = 0.1 (optimal noise parameter then becomes σ^2^ = 10^–4^). The train/test errors are 0.33/0.34 eV, and particularly in the visible region (corresponding to the PBE bandgaps up to about 2 eV), the perturbation makes the model nearly useless with errors on the order of 1 eV. With *A* = 0.01, one obtains train/set errors of 0.19/0.21 eV which are only slightly above those without perturbation (optimal noise parameter in this case is σ^2^ = 10^–6^). In this case, one can also conclude that limitations on prediction accuracy due to the data dominate those due to both the restricted shape of the activation function and its instabilities. The results are shown in Figure 8: at *A* = 0.01, the perturbations of the NAFs are practically invisible. Sufficiently stable circuits likely can be designed based on inorganic semiconductor transistors. In light of these results, based on available experimental reports,^20,54^ organic transistors still require improvements in stability for use in neural circuits. Moreover, organic transistors possess characteristics such as flexibility and large-area manufacturability and can facilitate the future development of low-energy computing and efficient logic chips based on NN computing. This suggests a promising direction of research effort.

**Figure 7:**
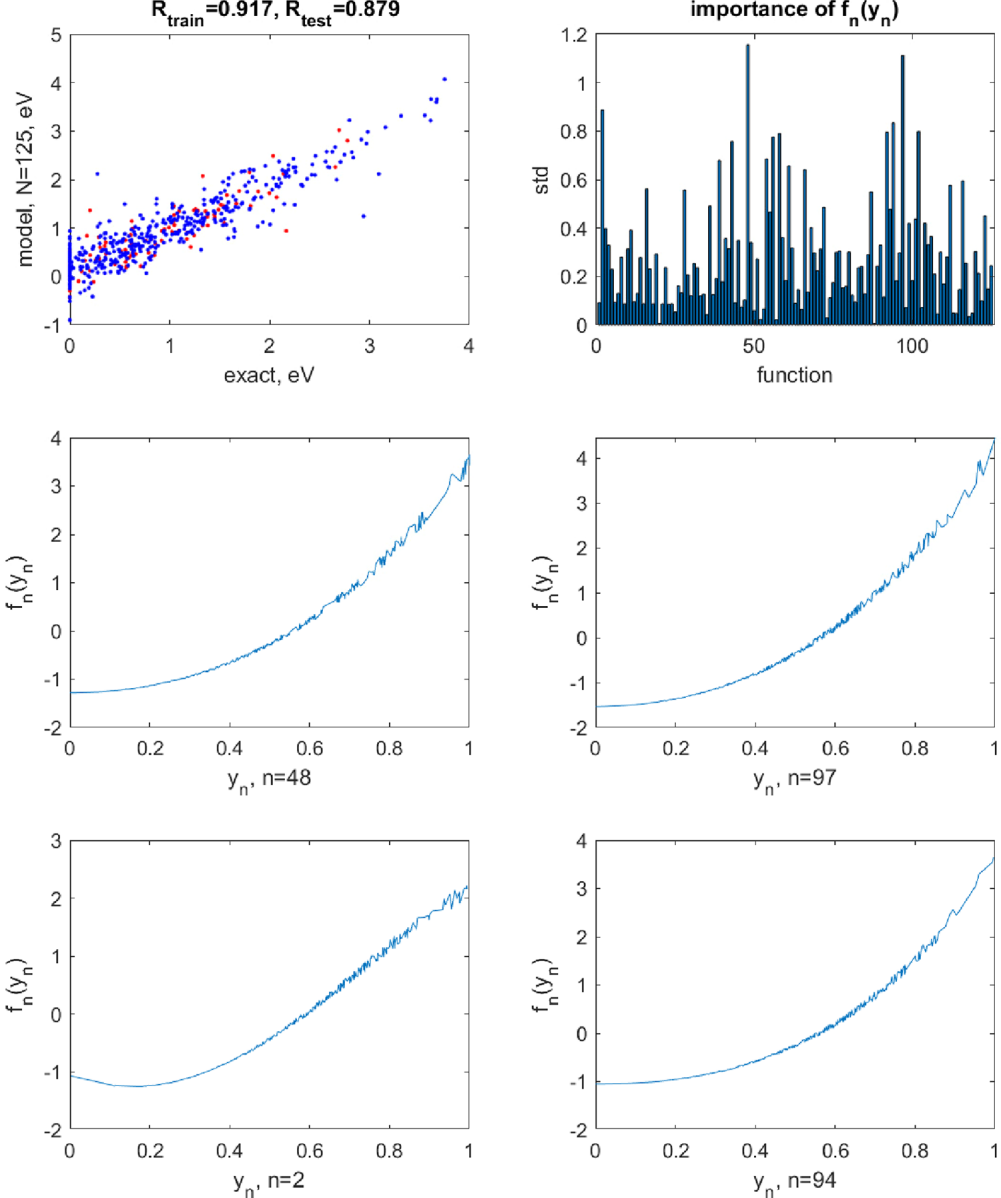
Same as Figure 6 but with the activation functions perturbed with *A* = 0.1.

**Figure 8:**
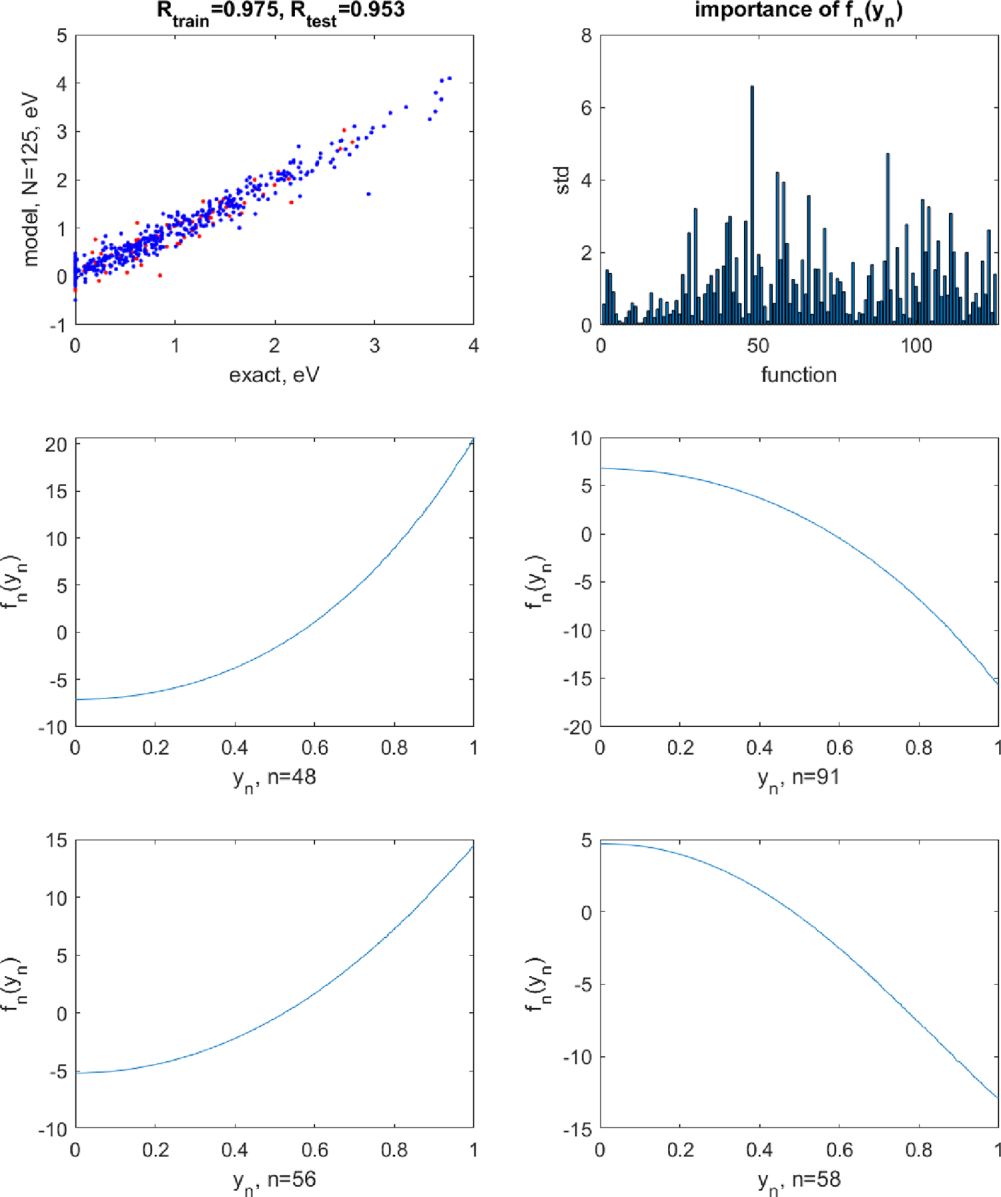
Same as Figure 6 but with the activation functions perturbed with *A* = 0.01.

In conclusion, we computationally investigated the performance of neural networks with neuron activation functions typical of transistors that could be used in dedicated analog NN circuits. Specifically, we investigated the influence of two factors: the limitation of the shapes of the neuron activation functions to those easily realizable with typical transistor transfer functions and instabilities of the NAF due to noise and other factors present in analog circuits. We restricted only the *type* of the shape of the NAFs but otherwise allowed NAFs to be different for different neurons, in the paradigm that a pretrained NN can be realized in silico, and as no further training (e.g., with backpropagation facilitated by identical neurons) would be required, there is no need to make all neurons identical. The performance of the restricted-shape activation functions was compared to that with optimal neural activation functions. We found that while the shape restriction leads to an accuracy penalty when highly accurate NNs are required (where desired and obtainable correlation between the exact and predicted values is on the order of 1.00, as in the interatomic potential example we considered), for many real-life applications, there expected to be no significant accuracy penalty (as in the example from materials informatics). On the other hand, we found that there is high sensitivity to the instability in the shape of the activation function, suggesting that high-precision circuitry should be developed, apparently beyond what has been reported for synaptic transistors to date. This suggests a promising direction for research on transistor-based neural circuits.

## Supporting Information Available

Matlab codes (.m files); data on which the calculations in the article were performed (ASCII.dat files); a README.txt ASCII file explaining the package and how it can be used (ZIP)
